# Relative and absolute intensity accelerometer metrics decipher the effects of age, sex, and occupation on physical activity

**DOI:** 10.1186/s12889-025-21800-w

**Published:** 2025-03-06

**Authors:** Fabian Schwendinger, Raphael Knaier, Jonathan Wagner, Denis Infanger, Eric Lichtenstein, Timo Hinrichs, Alex Rowlands, Arno Schmidt-Trucksäss

**Affiliations:** 1 Division of Sports and Exercise Medicine, Department of Sport, Exercise and Health, University of Basel, Basel, Switzerland; 2 Division of Movement and Exercise Science, Department of Sport, Exercise and Health, University of Basel, Basel, Switzerland; 3 Assessment of Movement Behaviours Group (AMBer), Diabetes Research Centre, Leicester General Hospital, University of Leicester, Gwendolen Rd, Leicester, UK; 4 National Institute for Health Research (NIHR) Leicester Biomedical Research Centre (BRC), University Hospitals of Leicester NHS Trust and the University of Leicester, Leicester, UK; 5 Alliance for Research in Exercise, Nutrition and Activity (ARENA), UniSA Allied Health and Human Performance, University of South Australia, Adelaide, Australia; 6 Department of Clinical Research, University Hospital Basel, Basel, Switzerland

**Keywords:** Accelerometry, Cardiorespiratory fitness, GGIR, Activity monitors

## Abstract

**Background:**

To investigate whether quantifying both the absolute and relative intensity of physical activity (PA) improves understanding of age, sex, and occupation-related differences in PA in healthy adults aged 20–89.

**Methods:**

In the cross-sectional COmPLETE study, participants (*N* = 460, 48% women, age 55 [IQR 37, 71]) wore accelerometers for up to 14 days and underwent cardiopulmonary exercise testing. Average acceleration (AvAcc) and distribution of intensity (IG) of PA across the day were expressed in absolute terms (__ABS_) and relative (__REL_) to the acceleration at the individual´s maximum intensity, predicted from cardiorespiratory fitness.

**Results:**

After initial increases, AvAcc__ABS_ and IG__ABS_ continuously declined beyond age 40–45, whereas AvAcc__REL_ and IG__REL_ increased until stabilising at age ~ 70 and declining at age ~ 60, respectively. Cardiorespiratory fitness constantly declined. Women had trivially higher AvAcc__ABS_ and moderately higher AvAcc__REL_, but not IG__ABS_ and IG__REL_, than men. Occupations involving at least moderate PA showed higher AvAcc__ABS_ and AvAcc__REL_, but not IG__ABS_ and IG__REL_ indicating longer periods of low-intensity PA, compared to sitting/standing occupations.

**Conclusions:**

Distinct age trajectories of absolute and relative metrics as well as cardiorespiratory fitness suggest that the age-related decline in the latter preceded that of PA. Women’s higher AvAcc__ABS_ and AvAcc__REL_ relate to more low-intensity PA combined with lower cardiorespiratory fitness rather than more health-enhancing higher-intensity PA. Finally, the intensity profile of occupational PA may provide insight into why occupational PA appears to lack a prophylactic association with health. Quantifying both the absolute and relative intensity of accelerometer-assessed PA provides greater insight than either alone.

**Trial registration:**

On clinicaltrials.gov (NCT03986892). Retrospectively registered 14 June 2019.

**Supplementary Information:**

The online version contains supplementary material available at 10.1186/s12889-025-21800-w.

## Introduction

Accelerometers have substantially advanced physical activity (PA) research by enabling the accurate and continuous measurement of human movement. This has important implications for PA surveillance and epidemiological research such as providing information on intensity and volume of PA, understanding patterns of inactivity, and allowing for a precise assessment of the relationship of PA and health outcomes [1–3]. While the use of accelerometers has been revolutionary for PA research, further potential value lies in effectively interpreting the raw data to derive meaningful insights on PA patterns.

Various metrics can be derived from raw accelerometer data to describe PA [4]. Aside from cut-point-based metrics (i.e. light, moderate, and vigorous PA in minutes), alternative cut-point-free metrics are increasingly being used in research [2, 3, 5–7]. Cut-point-free metrics have the advantage of enhancing comparability across studies, populations, and the most commonly used raw acceleration devices [8].

The average level of acceleration observed over each 24-h period assesses the average daily acceleration and is commonly used as a proxy for volume of PA (AvAcc__ABS_) [6, 7]. The distribution of the acceleration across the day (e.g. the relative proportions of time in higher and lower intensities of PA) provides a complementary measure of the PA profile, the intensity gradient, that gives independent information to total volume (IG__ABS_) [6, 7]. This is important as the combination of volume and intensity may explain more variance in health than either alone [6, 7].

Looking beyond the absolute intensity of accelerometer-based PA, Orme et al. [9] recently proposed expressing absolute metrics of PA relative to the measured or predicted acceleration corresponding to an individual’s physical capacity/cardiorespiratory fitness (CRF). This approach was recently used by Rowlands et al. [10] to develop the relative intensity gradient (IG__REL_), which reflects the intensity distribution of a 24-h day relative to an individual’s maximum (aerobic) acceleration. The authors demonstrated that individuals with low IG__REL_, i.e. they had spare capacity, were more likely to increase the absolute intensity of their free-living physical activity (IG__ABS_) following a PA intervention [10]. Relative accelerometer metrics may thus have potential to improve personalisation of PA interventions [10].

These developments also pave the way for thinking about the average acceleration across the day in relative terms. Expressing both the average daily acceleration (AvAcc__ABS_ & AvAcc__REL_) and the distribution of intensity (IG__ABS_ & IG__REL_) across the day in absolute and relative terms may expand our understanding of PA further and be helpful in research and clinical practice.

Both the volume and intensity of accelerometer-assessed PA decrease with age, [7, 11] a decline that is less pronounced in self-reported data [11]. It is also unclear whether the age trajectories of PA are moderated by the age-related decline in CRF. Likewise, observable sex-differences in PA differ between device-based and self-reported data [11, 12]. Given that self-reported data are influenced by relative intensity of PA, [13] leveraging relative alongside absolute accelerometer metrics might offer more nuanced insights into the association of PA with age and sex. Moreover, evidence suggests occupational PA does not confer the same health benefits as leisure-time PA [14, 15]. Relative accelerometer metrics might elucidate differences in potential underlying mechanisms such as intensity patterning and physiological load between varying occupation types, helping to understand the PA paradox [14]. For example, occupational PA may be at an insufficient relative intensity to lead to meaningful fitness adaptations and/or be sustained over long periods without the recovery periods that would enable meaningful adaptations [14].

Therefore, this study aimed to use a sample of healthy adults aged 20 to 89 years to investigate 1) the degree to which AvAcc__REL_, AvAcc__ABS_, IG__REL_, and IG__ABS_ explain a unique fraction of the variance in PA and 2) whether quantifying the average daily acceleration (AvAcc__ABS_ & AvAcc__REL_) and the distribution of intensity across the day (IG__ABS_ & IG__REL_) in absolute and relative terms improves understanding of differences in PA by age, sex, and occupation.

## Materials and methods

### Study design

Data collection took place as part of the population-based, cross-sectional COmPLETE cohort study at the Department of Sport, Exercise and Health at the University of Basel, Switzerland. COmPLETE aimed to determine trajectories of physical fitness components in a healthy population sample between ages 20 and 100. More details are available in the study protocol [16]. The study was approved by the Ethics Committee of North-western and Central Switzerland (EKNZ 2017–01451) and all procedures followed the Declaration of Helsinki. COmPLETE was registered on clinicaltrials.gov (NCT03986892).

### Study participants

Recruitment took place by sending unaddressed letters to randomly selected postal districts in the Basel area. Healthy adults ≥ 20 years of age, non-smoking, and with a body mass index (BMI) < 30 kg/m^2^ were eligible to partake in this study [16]. The presence of chronic exercise-limiting disease, known pregnancy or breastfeeding, orthopaedic problems hindering the examinations, inability to follow the study procedures, and contraindications for all-out exercise led to exclusion from the study [16]. Before participation, all subjects provided written informed consent. Details regarding the recruitment procedures are given in the study protocol [16].

### Study procedures

#### Anthropometric and occupation data

Body mass and fat percentage were determined by four-segment bioelectrical impedance analysis using the InBody 720 (Inbody Co. Ltd., Seoul, South Korea). The device is valid and reliable for estimating body fat mass [17, 18].

Occupational data were collected using the European Health Survey-Physical Activity Questionnaire (EHIS-PAQ) [19]. In the original item, occupation type is divided into four categories: no work-related PA, sitting and standing, walking or moderate work-related PA, and vigorous or physically demanding work-related PA [19]. All but two participants in the ‘no work-related PA’ category were retired (*n* = 151). The level of employment was self-reported on a categorical scale ranging from 0 to 100% of full-time employment in intervals of 10% [19].

#### Cardiopulmonary exercise testing

Respiratory gases were measured breath-by-breath during one out of five ramp protocols with linear increments of 7, 10, 15, 20, or 30 W/min on a cycle ergometer (Ergoselect 200; ergoline GmbH, Bitz, Germany) using the MetaMax 3B portable metabolic system (Cortex Biophysik GmbH, Leipzig, Germany) [16]. To ensure the test was performed maximal voluntary exertion, presence of an oxygen uptake (V̇O_2_) plateau was the primary criterion and respiratory exchange ratio as well as % of age-predicted maximum heart rate were the secondary criteria as detailed elsewhere [20]. The choice of protocol was based on the subject’s estimated CRF to yield an exercise duration between six and 18 min [16, 21]. The mean of the highest consecutive V̇O_2_values (30-s mean) was defined as V̇O_2peak_.

#### Accelerometer-based measurement of physical activity

GENEActiv Original (Activinsights, Activinsights Ltd., Kimbolton, UK) triaxial accelerometers were used. Devices were initialised with a sampling frequency of 50 Hz and participants were instructed to continuously wear the device on their non-dominant wrist for up to 14 consecutive days [16]. PA data were processed using the raw data files (.bin format) in the R-package GGIR version 2.9.0 [4, 22–24]. A post-calibration error > 10 milli-gravitational units (m*g*) led to exclusion of accelerometer files. Euclidean norm minus one *g* with negative values rounded to zero (ENMO) was the accelerometer metric used to derive the below-described outcomes [23]. It reflects the magnitude of dynamic acceleration corrected for gravity averaged over 5-s windows expressed in m*g *[10, 23]. In line with other studies using 24-h wear protocols, a valid day was defined as wear time ≥ 14 h [7, 25]. Moreover, at total of at least four valid weekdays (Monday to Friday) + one Saturday and one Sunday and wear data for each 15 min period of the 24 h cycle were required for inclusion in the statistical analyses [26]. These strict criteria were chosen to ensure data are representative of free-living PA which has been shown to differ between weekdays, Saturdays and Sundays in our cohort [26].

#### Accelerometer outcomes

**Fig. 1:**
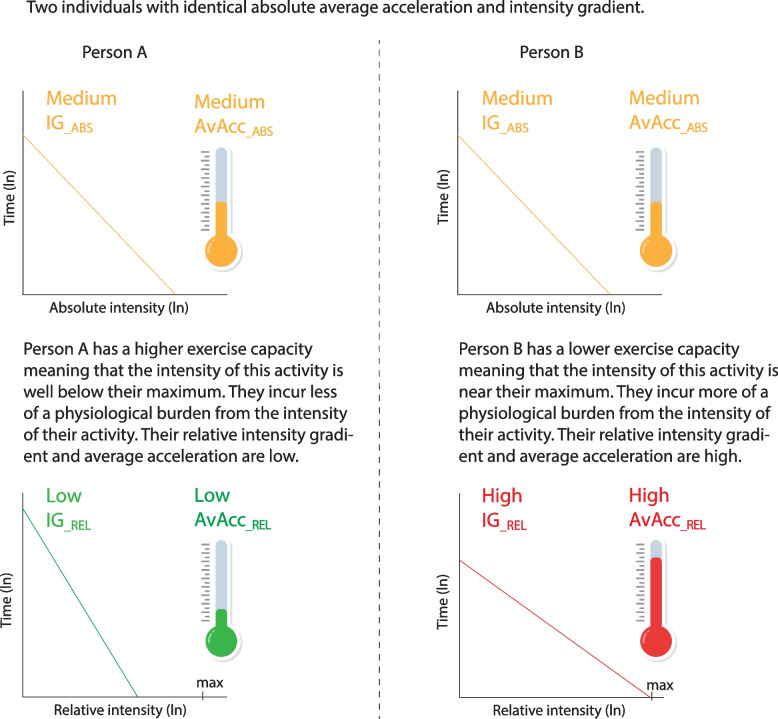
Comparison of absolute (__ABS_) and relative (__REL_) accelerometer metrics between two persons with identical absolute average acceleration (AvAcc) and intensity gradient (IG) but different exercise capacity. Modified from Rowlands et al. [10]

The following metrics were averaged across all valid days and refer to a 24-h cycle from midnight to midnight. Their corresponding names in GGIR outputs are available in Supplemental Digital Content (SDC) Table 1. The concept of all analytical metrics is illustrated in Fig. 1.

**Table 1:** Cohort characteristics, overall and stratified by sex

**Characteristic**	**Overall**, *N* = 460	**Male**, *N* = 238	**Female**, *N* = 222
Age, years	55 (37, 71) [20—89]	56 (37, 71) [20—89]	54 (36, 71) [20—89]
Body mass index, kg·m^−2^	23.5 (21.8, 25.4)	24.4 (22.7, 26.4)	22.5 (20.9, 24.4)
Body fat, %	23.0 (17.7, 28.9)	20.0 (15.1, 25.2)	26.9 (21.7, 33.0)
*Smoking status*			
Non-Smoker	364 (79%)	187 (79%)	177 (80%)
Ex-smoker > 10 years	95 (21%)	50 (21%)	45 (20%)
Unknown	1 (0%)	1 (0%)	0 (0%)
*Occupation type*			
At least moderate PA	132 (29%)	50 (21%)	82 (37%)
No work-related PA	82 (18%)	49 (21%)	33 (15%)
Sitting/standing	246 (53%)	139 (58%)	107 (48%)
AvAcc__ABS_, m*g*	30.1 (25.3, 36.7)	30.2 (24.9, 37.0)	29.9 (25.5, 36.0)
AvAcc__REL_, %	2.94 (2.42, 3.59)	2.71 (2.21, 3.15)	3.24 (2.67, 3.91)
IG__ABS_	−2.484 (−2.614, −2.331)	−2.441 (−2.581, −2.304)	−2.536 (−2.644, −2.368)
IG__REL_	−3.076 (−3.267, −2.921)	−3.085 (−3.284, −2.921)	−3.066 (−3.249, −2.921)
Peak oxygen uptake, mL·kg^−1^·min^−1^	34.4 (26.9, 42.4) [13.5—65.1]	37.9 (29.7, 46.0) [16.4—65.1]	30.6 (24.2, 37.8) [13.5—53.5]
Pred. maximum ENMO, m*g*	1′058.8 (824.2, 1′305.8)	1′165.4 (911.5, 1′418.2)	940.6 (741.2, 1′162.3)

Data are presented as median (25th, 75th percentile), [Range], or n (%). All variables are presented on their original scales

*Abbreviations: _*_*ABS*_ Absolute, *AvAcc* Average acceleration, *ENMO* Euclidean norm minus one, *IG* Intensity gradient, *PA* Physical activity, *_*_*REL*_ Relative

Absolute average acceleration (AvAcc__ABS_) is the arithmetic average of ENMO across a 24-h day in m*g*.

Relative average acceleration (AvAcc__REL_) is AvAcc__ABS_ expressed in relation to the predicted maximum (aerobic) acceleration (m*g*) of an individual. We predicted maximum acceleration based on the linear relationship of ENMO and oxygen uptake (V̇O_2_) [27]. Based on the assumption of the relationship between V̇O_2_ and ENMO being linear for aerobic activities and the regression model by Hildebrand et al. [27] (SDC Fig. 1), we calculated maximal ENMO corresponding to the individuals’ CRF (V̇O_2peak_ measured during cardiopulmonary exercise testing) by extrapolation. This is an estimate of the maximum aerobic acceleration an individual can produce. Further details are in the SDC.

Absolute intensity gradient (IG__ABS_) describes the intensity distribution of PA across a 24-h day. Time accumulated in incremental acceleration bins (bin size = 25 mg) is expressed in relation to the mid-point of each intensity bin [5, 10]. The slope of a linear regression including both log-transformed variables (to linearise the curvilinear relationship) is the IG of an individual [6, 10].

Relative intensity gradient (IG__REL_) describes the relative intensity distribution of PA across a 24-h day expressed in relation to an individual’s maximum (aerobic) acceleration (m*g*) (as described earlier) [27]. It is derived from the epoch-level comma-separated values (.csv) files generated by GGIR using the custom-built R script available at www.github.com/Maylor8/Relative-Intensity-Gradient [10]. Calculations are similar to that of IG__ABS_ only that log-transformed accumulated time is regressed on log-transformed relative intensity instead of absolute intensity [10]. The mid-percentage of relative intensity bins of 5% (5 to > 300% of maximum acceleration) is used in this case [10].

MX metrics reflect the acceleration (m*g*) above which a person’s most active X minutes are accumulated [28]. The relative MX metrics were calculated using the individual’s predicted maximum aerobic acceleration: MX / maximum acceleration * 100 [9, 10]. This was done to display the absolute and relative intensity of the PA profile of the cohort and selected subgroups (i.e. stratified by age groups, sex, and occupation type). MX plots were generated for periods of 1–720 min (12 h) both on the original scale and Z-transformed (to better visualise differences in longer periods) using customised open-source code available at: www.github.com/Maylor8/RadarPlotGenerator.

### Statistical analyses

Statistical analyses and figures were done in R version 4.2.0 [29]. Accelerometer metrics in text are presented as Z-scores unless stated otherwise.

#### Correlation analyses

Correlations between absolute and relative cut-point-free metrics were examined using Spearman’s Rank Correlation. A principal component analysis of AvAcc__REL_, AvAcc__ABS_, IG__REL_, and IG__ABS_, was conducted to investigate the degree of information overlap and unique information among all relative and absolute accelerometer metrics [30].

#### Regression analyses

The association between age, sex, occupation type and each of the absolute and relative cut-point-free metrics was examined using a series of multiple linear regression models. Dependent variables (AvAcc__REL_, AvAcc__ABS_, IG__REL_, IG__ABS_, and MX metrics) were Z-transformed for better comparability. A directed acyclic graph identified the minimal necessary parameters included in the models to estimate unbiased effects (Suppl. Fig. 2) [31]. Age (continuous variable), sex (dichotomous variable: female, male), and occupation type (categorical variable: at least moderate PA, no work-related PA, sitting/standing) were included as independent variables. Moreover, models were adjusted for body fat percentage (continuous variable), seasonality (categorical variable: spring, summer, autumn, or winter), and level of employment (categorical variable: not working, 10–50%, 60–90%, and 100% of full-time employment). We used third-degree polynomials to model all continuous independent variables [32]. Fulfilment of model assumptions was verified using residual diagnostics. Dependent variables were log-transformed before Z-transformation in case assumptions for normality distribution and homogeneity of residuals were not fulfilled. If our data indicated an association based on the estimate and the lower or upper limits of the 95% CI included zero (± 5% of the width of the interval), this was flagged accordingly. Effect sizes were categorised as small (≥ 0.1 to < 0.3), medium (≥ 0.3 to < 0.5) and large (≥ 0.5) on the Z-scale [33].

#### Sensitivity analyses

Due to the high number of retired individuals reporting ‘no work-related PA’, occupational analyses were re-run without retired individuals. Moreover, occupational analyses were re-run including only weekdays to examine potential difference in the strength of association compared to including all days.

## Results

Table 1 shows cohort characteristics stratified by sex. The flow of participants is presented in Suppl. Fig. 3. Of a total of 629 participants in the COmPLETE study, 460 were included in the final statistical analyses.

### Relationship between relative and absolute cut-point free accelerometer metrics

Details on the relationship between relative and absolute cut-point free accelerometer metrics are available in the SDC.

### Association of relative and absolute metrics with age

**Fig. 2:**
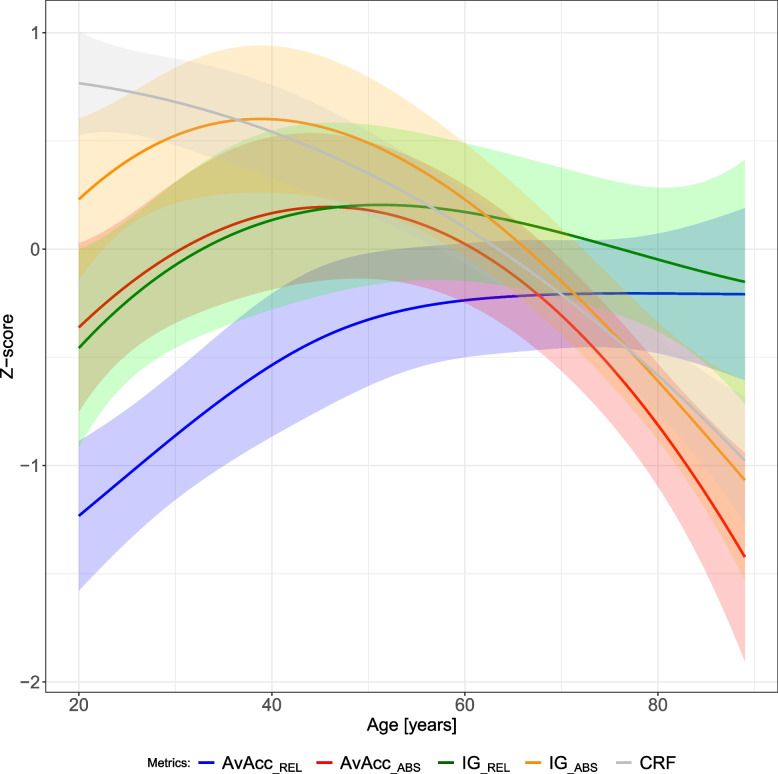
Association of relative and absolute cut-point-free accelerometer metrics (Z-transformed) with age. All models were adjusted for sex, occupation type, body fat percentage, seasonality, and level of employment. Age and body fat percentage were modelled using third-degree polynomials. Shading reflects 95% confidence interval. Abbreviation: CRF, cardiorespiratory fitness

The association of relative and absolute accelerometer metrics with age are compared to each other in Fig. 2. AvAcc__ABS_, IG__ABS,_ increased from age 20, plateauing briefly around age ~ 40. Beyond age 40–45, both AvAcc__ABS_ and IG__ABS_ continuously declined. In contrast, AvAcc__REL_ and IG__REL_ continued to increase before stabilising at age ~ 70 or declining at age ~ 60, respectively. Detailed results of the linear regression models are in Suppl. Table 2 (Z-transformed dependent variable), Suppl. Table 3 (original scales), and Suppl. Table 4 (in percentages).

**Fig. 3:**
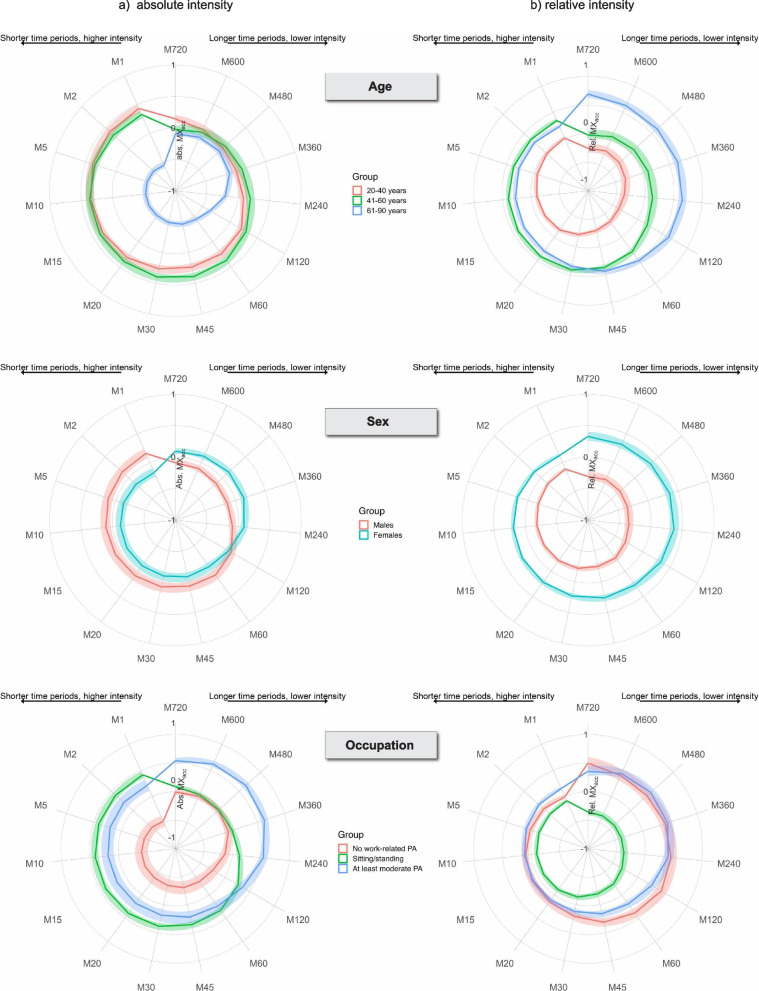
Adjusted Z-transformed MX plots for (**a**) absolute intensity and (**b**) relative intensity, stratified by age category (top row), sex (middle row), and occupation type (bottom row). The plot describes the minimum absolute (left) and relative intensities (right) that were accumulated in the most active periods in a 24-h day. Periods range (clockwise) from the most active 720 min (M720; 12 h) to the most active minute (M1). More extended shapes in certain directions indicate more intense physical activity during these periods. Metrics were adjusted for age (continuous) or sex (dichotomous) or occupation type (categorical), body fat percentage (continuous), seasonality (categorical), and level of employment (categorical). Continuous independent variables were modelled using third-degree polynomials. Shading reflects 95% confidence interval of the data. Plots on the original scale (adjusted and unadjusted) and without retired participants (only for occupation) are in the SDC (Suppl. Figs. 6–13)

The lower absolute intensity of the PA profile in the older participants was most evident during the most intense 60 min of the day, with a small to large association between age-group and the absolute MX metrics M1 to M60. However, in longer, less intense periods (M120 to M720) the associations ranged from small to trivial. For the latter, our data were also compatible with negligible to moderate effects (Fig. 3a, abs. MX; Suppl. Fig. 7). In contrast, relative intensity was lowest in the youngest age-group across all time periods (1 min to 12 h), with small to large between-group differences observed for all relative MX metrics depending on the age group (Fig. 3b, rel. MX; Suppl. Fig. 7).

### Association of relative and absolute metrics with sex

While AvAcc__ABS_ was only trivially associated with sex, being 5% higher in women than men (0.18, 95% −0.01 to 0.37), AvAccel__REL_ was 13% higher in women (0.45, 95% CI 0.27 to 0.64). Sex differences for the IG were negligible (IG__ABS_: −0.05, 95% −0.24 to 0.13; IG__REL_: 0.12, 95% −0.11 to 0.34). Detailed results of the regression models are in Suppl. Tables 2–4.

The trend towards a higher AvAcc__ABS_ over the PA profile in women was due to higher intensities during longer periods, M360 (6 h) to M480 (8 h) (Fig. 3a, abs. MX; Suppl. Fig. 8). In contrast, the higher AvAcc__REL_ was due to small to moderately higher relative intensity in women than in men across short as well as long time periods, M2 to M720 (12 h) (Fig. 3b, rel. MX; Suppl. Fig. 8).

### Association of relative and absolute metrics with occupation status

**Fig. 4:**
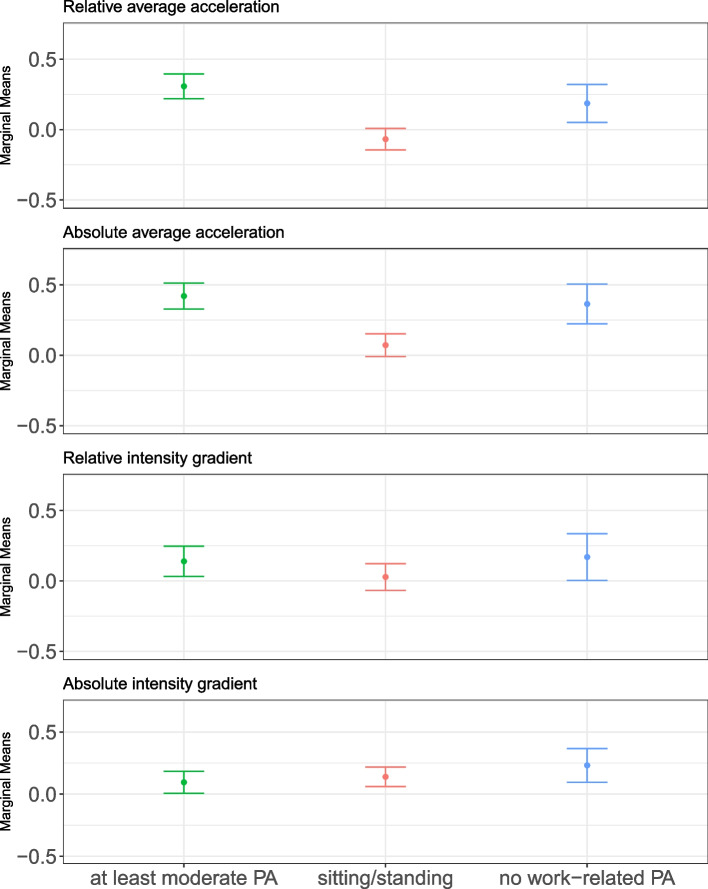
Association of relative and absolute cut-point-free accelerometer metrics with occupation type. Figure shows marginal means and standard errors (Z-score). All models were adjusted for sex, occupation type, body fat percentage, seasonality, and level of employment. Age and body fat percentage were modelled using third-degree polynomials. This analysis without retired participants is presented in Suppl. Fig. 6. Abbreviations: PA, physical activity

Occupation type was moderately associated with AvAcc__ABS_ and AvAcc__REL_ (Fig. 4; Suppl. Tables 2–4). Explicitly, predominantly sitting or standing at work was independently associated with lower AvAcc__ABS_ (−9.81%, 95% CI −15.42 to −4.2) and AvAcc__REL_ (−11.02%, 95% CI −16.6 to −5.44) compared to at least moderate work-related PA (Fig. 4). There was a negligible association of occupation type with IG__REL_ or IG__ABS_ (Fig. 4; Suppl. Table 2). Results were consistent when considering only weekdays (Suppl. Table 5). Considering only weekend days, associations of occupation type with AvAcc__ABS_ and AvAcc__REL_ were smaller and negligible (Suppl. Table 6).

The higher AvAcc__ABS_ over the PA profile in those with moderate work-related PA relative to those in sit/stand occupations was due predominantly to higher intensities during the longer periods, with small to moderate associations evident for M120 to M720 (12 h) (Fig. 3a; Suppl. Fig. 9).

Similarly, the higher AvAcc__REL_ in those with moderate work-related PA relative to those in sit/stand occupations was predominantly due to higher relative intensity during long periods of the day, with small to medium associations observed for M720 to M360 (Fig. 3b, Suppl. Fig. 9).

## Discussion

Our study demonstrated that 1) the relative intensity of PA increased with age while the absolute intensity plateaued. This, together with a continuously declining CRF, indicates the age-related decline in cardiorespiratory fitness might precede that of PA. 2) Women had trivially higher AvAcc__ABS_, yet moderately higher AvAcc__REL_. The higher average acceleration across the day (volume) was due to more time spent at lower intensity accelerations. However, in combination with the women’s lower CRF, this resulted in the greater sex difference in relative average acceleration across the day, rather than more of the health enhancing higher intensity of PA, as reflected in no difference in absolute IG. 3) Individuals with at least moderate occupational PA have higher AvAcc__ABS_ and AvAcc__REL_, but not IG. This occurs primarily through more intense longer periods, unlike leisure-time PA that tends to focus on shorter periods of health enhancing higher intensity PA (thus increases IG__ABS_ and IG__REL_) and allows time for recovery.

### Association of relative and absolute metrics with age

Up to 40 years of age, trajectories of AvAcc__ABS_ and IG__ABS_ indicate favourable changes with a higher absolute volume of PA in a day together with less time spent inactive and/or more time spent at higher absolute intensities of PA. Further, both relative parameters increased similarly, indicating that the increase in absolute intensity is reflected in an increase in physical effort. These trajectories were not apparent in a study of Finnish workers, where PA was assessed using heart rate sensors and relative intensity was calculated based on estimated CRF [34]. The researchers found a continuous decrease in moderate and vigorous PA with age and relatively stable trajectories of relative PA volume [34]. These differences might be explained by AvAcc__ABS_ being influenced by all intensities of PA, including light PA, different cohort characteristics, statistical analyses (continuous age data vs categorises), and methods (accelerometer and CPET vs heart rate and estimated CRF) [34].

Beyond 40 years of age, we documented a decrease in PA volume and a shift towards PA at lower absolute intensities and/or more inactive time, as shown previously [7]. However, the corresponding increase in AvAcc__REL_ shows that despite a lower volume of PA, it is accumulated at a rising percentage of an individual’s capacity (i.e., AvAcc__REL_). These trajectories of absolute and relative PA are in line with Kujala et al. [34]. Yet, it again should be considered that our findings reflect the full spectrum of PA while their study focused on moderate and vigorous PA.

**Fig. 5:**
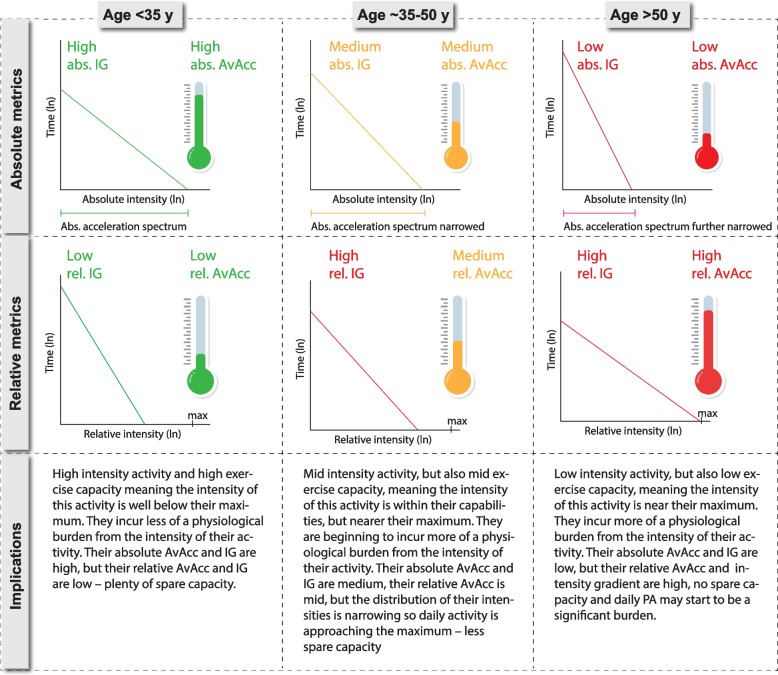
Illustration of how quantifying both absolute and relative intensity yields greater insights into physical activity patterns using the example of ageing

While AvAcc__REL_ increased, IG__REL_ stabilised. The dissimilarity in slopes between the two relative metrics indicates that they may reflect different information beyond 50 years of age. Assessing the relative IG alone, might lead to falsely assuming that daily PA evokes a similar physiological burden upon the individual despite advancing age. There is a narrowing of the absolute intensity spectrum attainable with advancing age due to the ageing-related decline in physical function [20, 35]. Activities of daily life that may have been on the lower end of the relative intensity spectrum with younger age (e.g., grocery shopping, household chores) may now require much more relative effort. Consequently, these types of activities take longer and make up for a larger fraction of the daily volume of PA, increasing the AvAcc__REL_ despite the lower absolute intensity (AvAcc__ABS_ and IG__ABS_) of the activities. This may suggest an increasing physiological burden across the day. However, the stability of the IG__REL_ post age ~ 40–45, before declining post age ~ 60, suggests that distribution of the more intense activity is adapted in accordance with declining capacity. This suggests that all four parameters are important to fully understand the ageing-related changes in PA patterns and age is an important variable to consider for personalising PA interventions and evaluation. A detailed illustration of how the four metrics interrelate with ageing is in Fig. 5.

MX plots further elucidate these age differences in absolute and relative metrics. While all age groups are similarly active at an absolute intensity for the longer duration MX metrics, the intensity of PA drops notably for the shorter periods of higher intensity in 61–90-year-olds compared to the younger individuals. Given the consistency in absolute MX metrics for 20–40 and 41–60-year-olds, the higher relative intensity of the MX metrics in 41–60-year-olds again suggests that the age-related decline in CRF precedes the decrease in PA intensity (Fig. 2). This also indicates that older adults are under a higher physiological load during long portions of the day compared to younger adults. Indeed, relative intensity seems to converge between middle-aged and older adults regarding shorter, more intense periods, despite the absolute intensity being much lower in the 61–90-year olds. We may hypothesise that while middle-aged individuals still engage in more intense activities, older individuals do not, but nevertheless they still experience the same higher physiological load due to lower CRF. This may be tackled by replacing inactive time with health-enhancing PA at an appropriate relative intensity to improve CRF and reduce daily physiological load.

### Association of relative and absolute metrics with sex

In our study, AvAcc__REL_ and AvAcc__ABS_, but not and IG__REL_ and IG__ABS_, tended to be higher in women. Higher AvAcc__ABS_, but not IG__ABS_, in women is consistent with UK Biobank, [36] but not with Mielke et al. [37] who noted no sex differences in AvAcc__ABS_ among Australian adults. However, the presence of sex differences may vary depending on geographical/societal context [38]. The greater difference in AvAcc__REL_ than AvAcc__ABS_ in women is explained by lower levels of CRF compared to men [20]. The greater volume in women was due to more time spent at lower intensity accelerations, rather than accumulating health enhancing higher intensity PA, as reflected in no difference in IG__ABS_.

### Association of relative and absolute metrics with occupation status

Individuals engaging in more physically demanding work exhibit higher average absolute and relative intensity of PA than individuals in more sedentary roles or those not engaging in work-related PA. This aligns with observations from the cross-sectional National Health and Nutrition Examination Survey (NHANES), which reported more weekday PA for individuals with active jobs than those with inactive jobs [39]. Our findings extend these by providing a relative PA perspective that is to date unique. Both the average absolute and relative intensity of PA is higher indicating that the higher relative intensity in individuals with more active jobs is due to them engaging in more intense PA rather than having a lower CRF. Confirming this, a sensitivity analysis showed no difference in V̇O_2peak_ between occupation types (data not presented). Our data suggests that higher relative intensity seen in those with greater absolute volume of PA is predominantly due to higher absolute intensities during longer periods (i.e., MX_acc_ > 120 min), explaining the lack of difference in IG by occupation in absolute or relative terms. Importantly, this also shows that despite their higher occupational PA, these workers do not have higher CRF.

These findings may have relevant implications for the PA paradox, [14] as they support the hypothesis that the patterns of PA associated with occupational PA may not be optimal for improving CRF or cardiovascular health, e.g., be of too low intensity (i.e. < 60% of maximum aerobic capacity) and/or too long duration, without appropriate recovery periods [14]. On the other hand, the average relative intensities of occupational PA over an 8-h working day may exceed the recommended levels for such longer periods (> 30%–35% of maximal aerobic capacity) [14, 40]. Moreover, the higher relative intensity during prolonged periods means more time spent at elevated heart rate, which is a known risk factor for cardiovascular disease and mortality [14, 41].

### Limitations

Predicted maximum acceleration, used to determine relative intensity of PA, is derived from the extrapolation of acceleration up to the value corresponding to V̇O_2peak_. This may induce inaccuracy compared to directly measuring maximum acceleration using ambulatory tests such as the incremental shuttle walking test [9]. However, we used the gold standard, cardiopulmonary exercise testing, to measure V̇O_2peak_ and our simulation showed good accuracy of the regression models by Hildebrand et al. [27]. Even if there may be a prediction error, using the same model to calculate maximum acceleration ensures comparability between participants within this study. PA patterns and the associations of accelerometer metrics with age, sex, and occupation type might differ between cohort characteristics, geographic and societal contexts. The findings of our study are hence primarily generalisable to a Central European healthy adult population. The categorisation by occupation type relied on self-reports, which were limited to a few categories and it is possible that some participants may not have been working during their monitoring period, potentially skewing the results. Yet, a sensitivity analysis showed consistent results when including only weekdays (Suppl. Table 5). Furthermore, the occupational aspect is primarily relevant for those still in the workforce. A considerable portion of our participants over the age of 65 years were retired (*n* = 153) and all except for two fell into the ‘no work-related PA’ category. Further, the main analyses were adjusted for age and level of employment in % of full-time. Moreover, subgroup analyses with retired participants excluded were similar (Suppl. Figs. 6 and10).

## Perspective

Our findings help decipher age, sex, and occupation type-related PA patterns by providing a perspective considering the individual´s physical capacity. Firstly, age-related changes in absolute and relative intensity indicated the age-related decline in CRF preceded a drop in absolute intensity and volume of PA. Despite increasing physiological load, absolute volume and intensity distribution of PA increased up to age 40 and declined thereafter. Secondly, the slightly higher PA volume in women is mainly explained by more time spent at lower intensity accelerations rather than higher (potentially) more health-enhancing intensity PA. This is accompanied by overall higher relative intensity due to lower CRF in women compared to men. Thirdly, we provided insights into hypotheses relating to the underlying mechanisms of the PA paradox. Higher occupational PA seems to increase absolute average acceleration of PA and thus physiological load, but is not accommodated by higher CRF, as indicated by higher relative intensity. The increased PA occurs primarily through higher intensities over longer periods of time, unlike leisure-time PA that focuses on shorter periods of health-enhancing PA at higher intensities and allows time for recovery. This knowledge may allow for better personalisation of PA interventions and interpretation of accelerometer-based PA data.

## Supplementary Information

Additional file 1. Supplemental digital content

## Data Availability

The data that support the findings of this study are available on request from the corresponding author. The data are not publicly available due to privacy or ethical restrictions.
