# Supplementary material for: Relative and absolute intensity accelerometer metrics decipher the effects of age, sex, and occupation on physical activity
Source: BMC Public Health. 2025 Mar 6;25:885. doi: 10.1186/s12889-025-21800-w (PMC11884089; doi:10.1186/s12889-025-21800-w)
Supplement: Supplementary file 1 — Additional file 1. Supplemental digital content [file 12889_2025_21800_MOESM1_ESM.docx]

**Supplemental Digital Content**

**Prediction of maximum (aerobic) acceleration (m*g*)**

Hildebrand and colleagues(1) simultaneously measured acceleration (processed to ENMO) and V̇O_2_ of various standardised activities and modelled a linear relationship between the two parameters. The regression equation for the wrist-worn GENEActiv was: V̇O_2_ in mL^.^kg^-1.^min^-1^ = 0.0323 m*g* + 7.49 (95% CI for alpha: 0.0321 to 0.0325, 95% CI for beta: 7.43 to 7.54, R^2^ = 76%).(1) Based on this information, and the V̇O_2_ and ENMO values (in m*g*) described, 1000 simulations (n = 900 per simulation, see Suppl. Figure 1) were computed. They produced a prediction interval of 1.74 mL^.^kg^-1.^min^-1^ and the mean absolute error was 0.70 [95% CI: 0.67; 0.74] mL^.^kg^-1.^min^-1^.


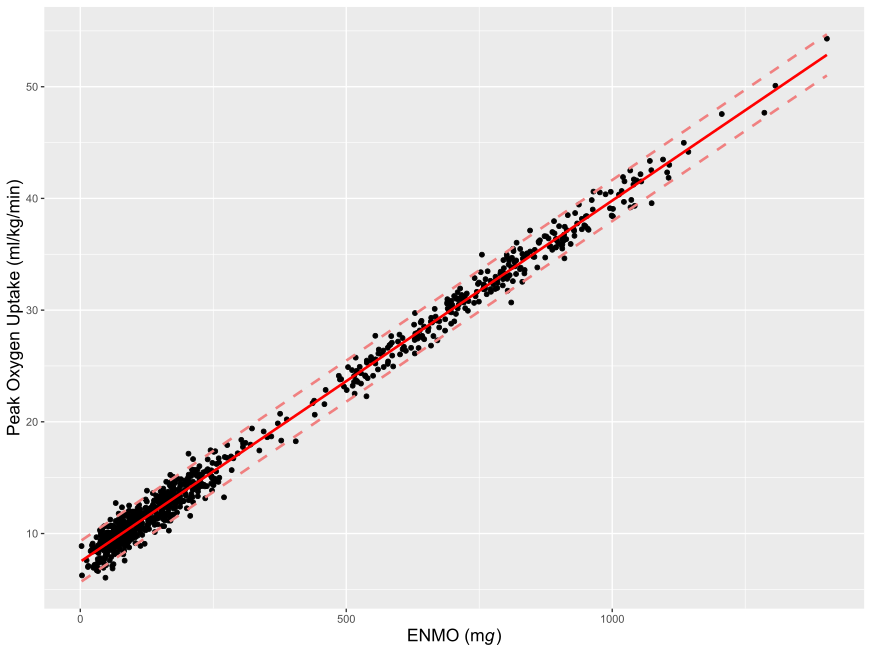


**Supplementary Figure 1.** Result of simulation based on regression of oxygen uptake and Euclidean norm minus one (ENMO) in Hildebrand et al.(1). Dashed lined represent the 95% prediction interval (width = 1.74 mL^.^kg^-1.^min^-1^).

**GGIR variable names**

All GGIR variables used in the analyses are reported below.

| Supplementary Table 1. GGIR variables used in the analyses and corresponding naming in the manuscript. | | | |
| --- | --- | --- | --- |
|  | **Name in manuscript** | **Name in GGIR(2)** | **Definition** |
| Part 2 | Intensity gradient | AD_ig_gradient_ENMO_0.24hr | Intensity distribution of physical activity across a 24-h day. A higher IG indicates less time spent at low intensity and/or more time spent at higher intensity.(3) |
|  | Average intensity | AD_mean_ENMO_mg_0.24hr | Arithmetic average of Euclidean norm minus one (ENMO) across a 24-h day in m*g* (proxy of physical activity volume).(3) |
|  | MX_acc_ (m*g*) | e.g. ‘AD_p50_ENMO_mg_0.24hr’ and ‘AD_p99.93056_ENMO_mg_0.24hr’ where p50 equals the 50^th^ %ile of the 24 h range, i.e. M720 (12 h) and p99.93056 equals the 99.93056^th^ %ile of the 24 h range, i.e. M1 (1 min) | MX metrics reflect the minimum acceleration in m*g* for the most active accumulated period of a specific length X. X is the number of minutes, i.e. 720min or 1 min)(4) |

**
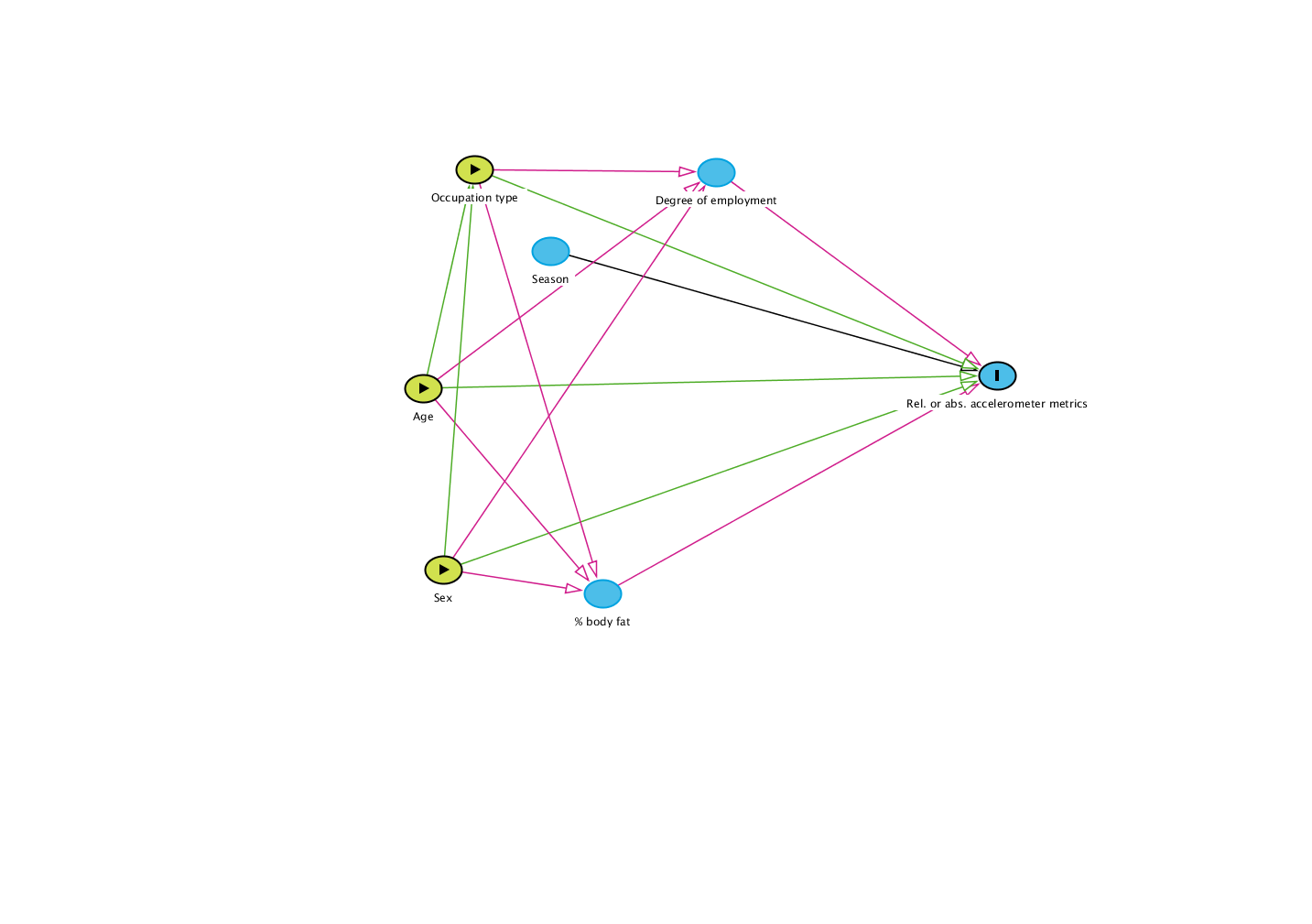
**

**Supplementary Figure 2.** Directed acyclic graph(5) to identify the minimal sufficient adjustment sets for estimating causal effects of age, sex, and occupation type on relative and absolute accelerometer metrics.

**
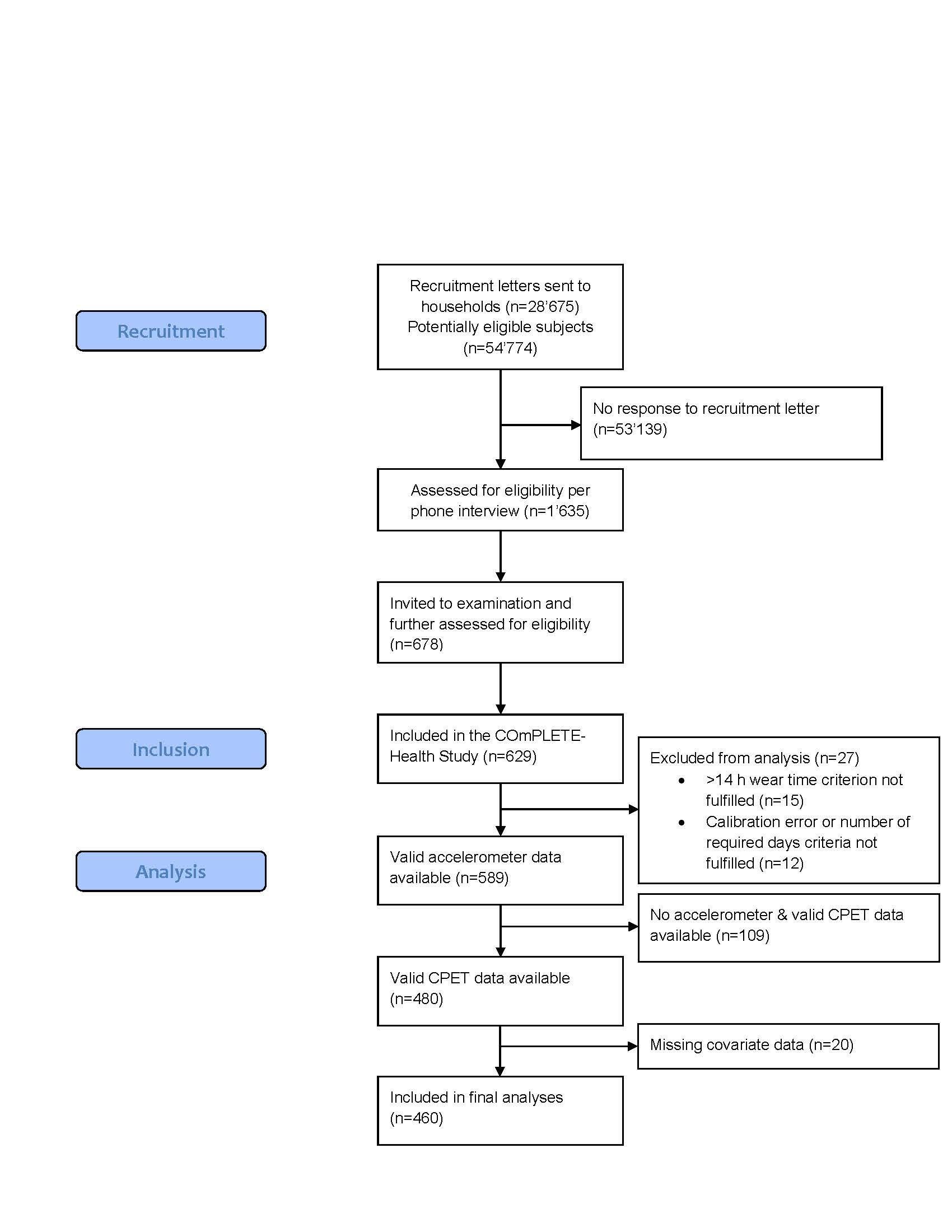
**

**Supplementary Figure 3.** Flow of study participants.

*Relationship between relative and absolute cut-point free accelerometer metrics*

All relative and absolute cut-point-free accelerometer metrics were moderately and positively correlated except for AvAcc__REL_ and IG__ABS_ (Suppl. Figure 4A). The results of the principal component analysis indicate that AvAcc__REL_ and IG__ABS_ contribute via a different dimension to explaining the variance in the data, whereas AvAcc__ABS_ and IG__REL_ contribute rather similar information to the overall variance (Suppl. Figure 4B).


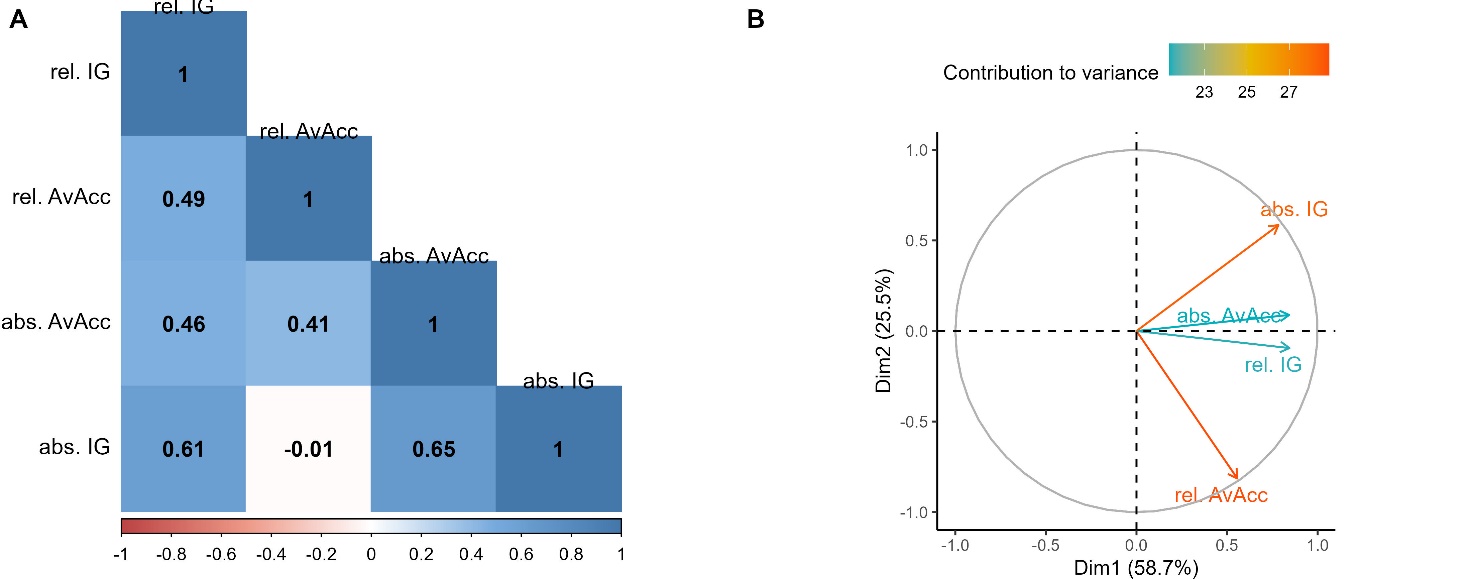


**Supplementary Figure 4.** A) Spearman correlation plot illustrating the relationship between relative and absolute cut-point-free accelerometer metrics. Darker colours indicate stronger correlations. B) Plot of principal component analysis showing the contribution of the respective parameters to the variance in the data. Abbreviations: AvAcc, average intensity; IG, intensity gradient.

*Association of absolute and relative metrics with age, sex, and occupation status*

| Supplementary Table 2. Regression models (estimate [95% confidence interval]) of the association between relative or absolute cut-point-free accelerometer metrics (Z-transformed) and sex, age, and occupation status. | | | | |  |  |
| --- | --- | --- | --- | --- | --- | --- |
| Parameter | **Abs AvAcc** | **Rel. AvAcc** | **Abs. IG** | **Rel. IG** |  |  |
| (Intercept) | 1.28  [0.56 to 1.99] | -0.6  [-1.28 to 0.09] | 0.74  [0.05 to 1.42] | -0.46  [-1.29 to 0.38] |  |  |
| Female sex | 0.18  [-0.01 to 0.37] | 0.45  [0.27 to 0.64] | -0.05  [-0.24 to 0.13] | 0.12  [-0.11 to 0.34] |  |  |
| *Age* |  |  |  |  |  |  |
| Segment 1 20-44 years | 0.98  [-0.1 to 2.07] | 1.07  [0.03 to 2.11] | 0.96  [-0.09 to 2] | 1.11  [-0.16 to 2.39] |  |  |
| Segment 2 45-65 years | 0.68  [0.02 to 1.35] | 1.14  [0.5 to 1.78] | -0.1  [-0.74 to 0.54] | 0.54  [-0.25 to 1.32] |  |  |
| Segment 3 66-89 years | -1.06  [-1.68 to -0.44] | 1.02  [0.43 to 1.62] | -1.3  [-1.89 to -0.7] | 0.31  [-0.42 to 1.03] |  |  |
| *Occupation type* |  |  |  |  |  |  |
| No work-related PA | -0.06 [-0.33 to 0.22] | -0.12  [-0.38 to 0.14] | 0.14  [-0.13 to 0.4] | 0.03  [-0.29 to 0.35] |  |  |
| Sitting/standing | -0.35 [-0.55 to -0.15] | -0.38 [-0.57 to -0.19] | 0.04  [-0.15 to 0.24] | -0.11  [-0.34 to 0.12] |  |  |
| *Degree of employment* | | | | |  | *Degree of employment* |
| 10-50% | -0.07  [-0.41 to 0.26] | 0.06  [-0.26 to 0.39] | -0.11  [-0.43 to 0.21] | 0  [-0.39 to 0.4] |  |  |
| 60-90% | -0.1  [-0.38 to 0.18] | 0.02  [-0.25 to 0.29] | 0.09  [-0.18 to 0.36] | 0.11  [-0.22 to 0.44] |  |  |
| 100% | -0.23  [-0.47 to 0.02] | -0.11  [-0.34 to 0.12] | 0.07  [-0.16 to 0.31] | 0.05  [-0.23 to 0.33] |  |  |
| *Season* |  |  |  |  |  |  |
| Spring | 0.19  [-0.03 to 0.4] | -0.06  [-0.27 to 0.15] | 0.33  [0.13 to 0.54] | 0.3  [0.04 to 0.55] |  |  |
| Summer | 0.28  [0.04 to 0.51] | 0.2  [-0.03 to 0.43] | 0.22  [-0.01 to 0.44] | 0.21  [-0.07 to 0.49] |  |  |
| Winter | -0.23  [-0.48 to 0.02] | -0.35  [-0.6 to -0.11] | 0.25  [0 to 0.49] | 0.06  [-0.24 to 0.35] |  |  |
| *Body fat percentage* | | | | |  | *Body fat percentage* |
| Segment 1 | -1.51  [-2.94 to -0.08] | -0.8  [-2.16 to 0.56] | -0.83  [-2.2 to 0.54] | -0.29  [-1.96 to 1.38] |  |  |
| Segment 2 | -1.66  [-2.4 to -0.92] | 0.12  [-0.58 to 0.82] | -1.41  [-2.12 to -0.71] | -0.45  [-1.31 to 0.41] |  |  |
| Segment 3 | -1.8  [-2.79 to -0.82] | 0.37  [-0.57 to 1.31] | -1.35  [-2.29 to -0.4] | -0.22  [-1.37 to 0.94] |  |  |
| Abbreviations: AvAcc, average intensity; IG, intensity gradient. | | | | |  |  |

| Supplementary Table 3. Regression models (estimate [95% confidence interval], on the original scale) of the association between relative or absolute cut-point-free accelerometer metrics and sex, age, and occupation status. | | | | |
| --- | --- | --- | --- | --- |
| Parameter | **Abs AvAcc (m*g*)** | **Rel. AvAcc (%)** | **Abs. IG** | **Rel. IG** |
| (Intercept) | 3.78  [3.58 to 3.98] | 0.91  [0.71 to 1.11] | -2.31  [-2.46 to -2.16] | -3.21  [-3.45 to -2.97] |
| Female sex | 0.05  [0 to 0.1] | 0.13  [0.08 to 0.19] | -0.01  [-0.05 to 0.03] | 0.03  [-0.03 to 0.1] |
| *Age* |  |  |  |  |
| Segment 1 20-44 years | 0.28  [-0.03 to 0.58] | 0.31  [0.01 to 0.62] | 0.21  [-0.02 to 0.44] | 0.32  [-0.05 to 0.68] |
| Segment 2 45-65 years | 0.19  [0.01 to 0.38] | 0.33  [0.15 to 0.52] | -0.02  [-0.16 to 0.12] | 0.15  [-0.07 to 0.37] |
| Segment 3 66-89 years | -0.3  [-0.47 to -0.12] | 0.3  [0.13 to 0.47] | -0.28  [-0.41 to -0.15] | 0.09  [-0.12 to 0.29] |
| *Occupation type* |  |  |  |  |
| No work-related PA | -0.02  [-0.09 to 0.06] | -0.04  [-0.11 to 0.04] | 0.03  [-0.03 to 0.09] | 0.01  [-0.08 to 0.1] |
| Sitting/standing | -0.1  [-0.15 to -0.04] | -0.11  [-0.17 to -0.05] | 0.01  [-0.03 to 0.05] | -0.03  [-0.1 to 0.03] |
| *Degree of employment* | | | | |
| 10-50% | -0.02  [-0.12 to 0.07] | 0.02  [-0.08 to 0.11] | -0.02  [-0.09 to 0.05] | 0  [-0.11 to 0.11] |
| 60-90% | -0.03  [-0.11 to 0.05] | 0.01  [-0.07 to 0.09] | 0.02  [-0.04 to 0.08] | 0.03  [-0.06 to 0.13] |
| 100% | -0.06  [-0.13 to 0] | -0.03  [-0.1 to 0.04] | 0.02  [-0.03 to 0.07] | 0.01  [-0.07 to 0.1] |
| *Season* |  |  |  |  |
| Spring | 0.05  [-0.01 to 0.11] | -0.02  [-0.08 to 0.04] | 0.07  [0.03 to 0.12] | 0.08  [0.01 to 0.16] |
| Summer | 0.08  [0.01 to 0.14] | 0.06  [-0.01 to 0.13] | 0.05  [0 to 0.1] | 0.06  [-0.02 to 0.14] |
| Winter | -0.06  [-0.14 to 0.01] | -0.1  [-0.18 to -0.03] | 0.05  [0 to 0.11] | 0.02  [-0.07 to 0.1] |
| *Body fat percentage* | | | | |
| Segment 1 | -0.43  [-0.83 to -0.02] | -0.24  [-0.64 to 0.16] | -0.18  [-0.48 to 0.12] | -0.08  [-0.56 to 0.39] |
| Segment 2 | -0.47  [-0.67 to -0.26] | 0.04  [-0.17 to 0.24] | -0.31  [-0.46 to -0.15] | -0.13  [-0.37 to 0.12] |
| Segment 3 | -0.51  [-0.78 to -0.23] | 0.11  [-0.17 to 0.38] | -0.3  [-0.5 to -0.09] | -0.06  [-0.39 to 0.27] |
| Abbreviations: AvAcc, average intensity; IG, intensity gradient. | | | | |

| Supplementary Table 4. Regression models (estimate [95% confidence interval], in percent) of the association between relative or absolute cut-point-free accelerometer metrics and sex, age, and occupation status. | | | | |  |
| --- | --- | --- | --- | --- | --- |
| Parameter | **Abs AvAcc** | **Rel. AvAcc** | **Abs. IG** | **Rel. IG** | |
| (Intercept) | 35.99  [15.9 to 56.08] | -17.48  [-37.48 to 2.52] | 16.14  [1.14 to 31.13] | -12.97  [-36.78 to 10.85] | |
| Female sex | 5.06  [-0.37 to 10.48] | 13.28  [7.88 to 18.67] | -1.12  [-5.17 to 2.93] | 3.35  [-3.08 to 9.77] | |
| *Age* |  |  |  |  | |
| Segment 1 20-44 years | 27.66  [-2.95 to 58.28] | 31.4  [0.93 to 61.88] | 20.94  [-1.91 to 43.79] | 31.68  [-4.62 to 67.97] | |
| Segment 2 45-65 years | 19.27  [0.5 to 38.03] | 33.44  [14.76 to 52.11] | -2.17  [-16.17 to 11.84] | 15.25  [-6.99 to 37.49] | |
| Segment 3 66-89 years | -29.86  [-47.29 to -12.44] | 30.08  [12.73 to 47.43] | -28.41  [-41.42 to -15.4] | 8.71  [-11.95 to 29.37] | |
| *Occupation type* |  |  |  |  | |
| No work-related PA | -1.58  [-9.29 to 6.12] | -3.55  [-11.22 to 4.11] | 2.98  [-2.76 to 8.73] | 0.86  [-8.27 to 9.99] | |
| Sitting/standing | -9.81  [-15.42 to -4.2] | -11.02  [-16.6 to -5.44] | 0.97  [-3.22 to 5.15] | -3.16  [-9.81 to 3.49] | |
| *Degree of employment* | | | | | |
| 10-50% | -2.06  [-11.54 to 7.41] | 1.9  [-7.53 to 11.34] | -2.4  [-9.47 to 4.68] | 0.03  [-11.21 to 11.26] | |
| 60-90% | -2.75  [-10.67 to 5.18] | 0.62  [-7.27 to 8.52] | 1.98  [-3.94 to 7.9] | 3.12  [-6.28 to 12.52] | |
| 100% | -6.37  [-13.19 to 0.45] | -3.25  [-10.04 to 3.54] | 1.61  [-3.48 to 6.7] | 1.43  [-6.65 to 9.52] | |
| *Season* |  |  |  |  | |
| Spring | 5.25  [-0.89 to 11.38] | -1.67  [-7.78 to 4.44] | 7.32  [2.74 to 11.9] | 8.45  [1.17 to 15.72] | |
| Summer | 7.75  [1.06 to 14.45] | 5.86  [-0.8 to 12.52] | 4.72  [-0.27 to 9.72] | 5.92  [-2.01 to 13.85] | |
| Winter | -6.47  [-13.6 to 0.66] | -10.4  [-17.5 to -3.3] | 5.43  [0.1 to 10.75] | 1.61  [-6.84 to 10.07] | |
| *Body fat percentage* | | | | | |
| Segment 1 | -42.55  [-82.72 to -2.39] | -23.56  [-63.54 to 16.42] | -18.13  [-48.1 to 11.85] | -8.22  [-55.83 to 39.39] | |
| Segment 2 | -46.73  [-67.47 to -26] | 3.55  [-17.09 to 24.19] | -30.97  [-46.45 to -15.5] | -12.79  [-37.37 to 11.79] | |
| Segment 3 | -50.71  [-78.41 to -23.01] | 10.91  [-16.66 to 38.48] | -29.5  [-50.17 to -8.83] | -6.15  [-38.98 to 26.69] | |
| Abbreviations: AvAcc, average intensity; IG, intensity gradient. | | | | |  |

| Supplementary Table 5. Regression models (estimate [95% confidence interval]) of the association between relative or absolute cut-point-free accelerometer metrics and sex, age, and occupation status for weekdays only (Z-transformed). | | | | |
| --- | --- | --- | --- | --- |
| Parameter | **Abs AvAcc** | **Rel. AvAcc** | **Abs. IG** | **Rel. IG** |
| (Intercept) | 1.22  [0.48 to 1.95] | -0.41  [-1.15 to 0.32] | 0.75  [0.05 to 1.45] | -0.55  [-1.39 to 0.28] |
| Female sex | 0.11  [-0.09 to 0.31] | 0.26  [0.06 to 0.46] | -0.07  [-0.26 to 0.11] | 0.06  [-0.16 to 0.29] |
| *Age* |  |  |  |  |
| Segment 1 20-44 years | 1.26  [0.15 to 2.38] | 0.16  [-0.96 to 1.28] | 0.89  [-0.17 to 1.95] | 0.93  [-0.34 to 2.2] |
| Segment 2 45-65 years | 0.47  [-0.21 to 1.16] | 0.72  [0.04 to 1.41] | -0.39  [-1.04 to 0.26] | 0.7  [-0.08 to 1.48] |
| Segment 3 66-89 years | -0.93  [-1.57 to -0.3] | 1.17  [0.54 to 1.81] | -1.34  [-1.94 to -0.74] | 0.18  [-0.54 to 0.91] |
| *Occupation type* |  |  |  |  |
| No work-related PA | -0.08  [-0.37 to 0.2] | 0.05  [-0.23 to 0.33] | 0.19  [-0.08 to 0.45] | 0.11  [-0.21 to 0.43] |
| Sitting/standing | -0.4  [-0.61 to -0.2] | -0.26  [-0.47 to -0.06] | 0.08  [-0.12 to 0.27] | -0.05  [-0.28 to 0.19] |
| *Degree of employment* | | | | |
| 10-50% | -0.09  [-0.43 to 0.26] | -0.08  [-0.43 to 0.26] | -0.14  [-0.46 to 0.19] | -0.01  [-0.4 to 0.39] |
| 60-90% | -0.06  [-0.35 to 0.23] | -0.32  [-0.61 to -0.03] | 0.18  [-0.1 to 0.45] | 0.21  [-0.12 to 0.54] |
| 100% | -0.37  [-0.62 to -0.12] | -0.29  [-0.54 to -0.04] | 0.09  [-0.14 to 0.33] | 0.07  [-0.21 to 0.35] |
| *Season* |  |  |  |  |
| Spring | 0.22  [0 to 0.45] | -0.22  [-0.45 to 0] | 0.36  [0.15 to 0.57] | 0.29  [0.03 to 0.54] |
| Summer | 0.26  [0.02 to 0.5] | -0.06  [-0.31 to 0.18] | 0.26  [0.02 to 0.49] | 0.23  [-0.04 to 0.51] |
| Winter | -0.16  [-0.42 to 0.1] | -0.39  [-0.65 to -0.13] | 0.32  [0.08 to 0.57] | 0.12  [-0.18 to 0.41] |
| *Body fat percentage* | | | | |
| Segment 1 | -1.47  [-2.94 to -0.01] | 0.37  [-1.1 to 1.84] | -1  [-2.39 to 0.4] | -0.41  [-2.08 to 1.26] |
| Segment 2 | -1.51  [-2.26 to -0.75] | 0.22  [-0.54 to 0.98] | -1.21  [-1.93 to -0.5] | -0.19  [-1.05 to 0.67] |
| Segment 3 | -1.65  [-2.67 to -0.64] | 0.7  [-0.31 to 1.71] | -1.39  [-2.35 to -0.43] | -0.18  [-1.33 to 0.97] |
| Abbreviations: AvAcc, average intensity; IG, intensity gradient. | | | | |

| Supplementary Table 6. Regression models (estimate [95% confidence interval]) of the association between relative or absolute cut-point-free accelerometer metrics and sex, age, and occupation status for weekend days only (Z-transformed). | | | | |
| --- | --- | --- | --- | --- |
| Parameter | **Abs AvAcc** | **Rel. AvAcc** | **Abs. IG** | **Rel. IG** |
| (Intercept) | 1.2  [0.49 to 1.91] | -0.48  [-1.27 to 0.3] | 0.66  [-0.06 to 1.39 ] | -0.04  [-0.87 to 0.79] |
| Female sex | 0.27  [0.08 to 0.46] | 0.32  [0.1 to 0.53] | 0.01  [-0.19 to 0.21 ] | 0.19  [-0.04 to 0.41] |
| *Age* |  |  |  |  |
| Segment 1 20-44 years | 0.43  [-0.65 to 1.52] | 0.05  [-1.14 to 1.25] | 0.73  [-0.38 to 1.83 ] | 1.1  [-0.17 to 2.36] |
| Segment 2 45-65 years | 0.94  [0.28 to 1.61] | 0.95  [0.21 to 1.68] | 0.57  [-0.11 to 1.25 ] | -0.02  [-0.8 to 0.75] |
| Segment 3 66-89 years | -1.07  [-1.69 to -0.45] | 0.49  [-0.19 to 1.17] | -0.93  [-1.56 to -0.3 ] | 0.47  [-0.25 to 1.19] |
| *Occupation type* |  |  |  |  |
| No work-related PA | 0.01  [-0.26 to 0.28] | -0.08  [-0.38 to 0.22] | 0.04  [-0.24 to 0.32] | -0.14  [-0.46 to 0.18] |
| Sitting/standing | -0.18  [-0.37 to 0.02] | -0.14  [-0.35 to 0.08] | -0.03  [-0.23 to 0.17] | -0.2  [-0.43 to 0.03] |
| *Degree of employment* | | | | |
| 10-50% | -0.01  [-0.34 to 0.33] | -0.09  [-0.46 to 0.28] | -0.06  [-0.4 to 0.29] | 0  [-0.39 to 0.39] |
| 60-90% | -0.06  [-0.34 to 0.22] | 0.21  [-0.1 to 0.52] | -0.13  [-0.42 to 0.15] | -0.15  [-0.48 to 0.18] |
| 100% | 0.11  [-0.13 to 0.35] | 0.12  [-0.15 to 0.38] | 0  [ -0.24 to 0.25] | -0.02  [-0.3 to 0.26] |
| *Season* |  |  |  |  |
| Spring | 0.08  [-0.13 to 0.3] | -0.25  [-0.49 to -0.01] | 0.21  [-0.01 to 0.43] | 0.17  [-0.08 to 0.43] |
| Summer | 0.26  0.03 to 0.5] | 0.13  [-0.13  to  0.4] | 0.11  [-0.13 to 0.35] | 0.06  [-0.22 to 0.33] |
| Winter | -0.32  [-0.57 to -0.07] | -0.35  [-0.63 to -0.07] | 0.08  [-0.18 to 0.34] | -0.11  [-0.4 to 0.19 |
| *Body fat percentage* | | | | |
| Segment 1 | -1.57  [-2.99 to -0.15] | -0.11  [-1.68 to 1.46] | -0.39  [-1.84 to 1.06] | 0.04  [-1.62 to 1.7] |
| Segment 2 | -1.75  [-2.48 to -1.01] | 0.03  [-0.78 to 0.84] | -1.69  [-2.44 to -0.94] | -0.83  [-1.69 to 0.03] |
| Segment 3 | -1.88  [-2.86 to -0.9] | 0.38  [-0.7 to 1.46] | -1.01  [-2.01 to -0.01] | -0.22  [-1.37 to 0.92] |
| Abbreviations: AvAcc, average intensity; IG, intensity gradient. | | | | |

*
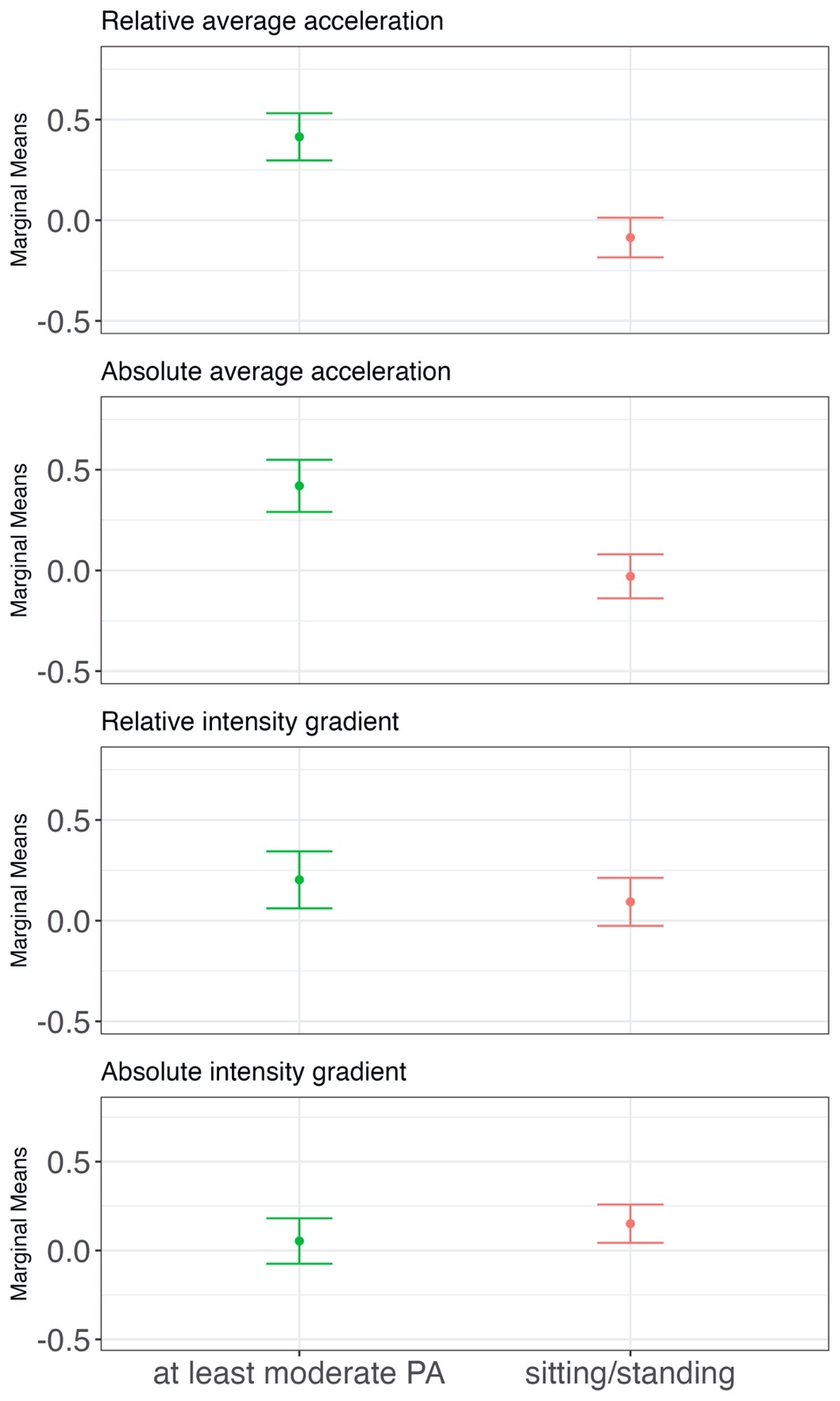
*

**Supplemental Figure 6.** Association of relative and absolute cut-point-free accelerometer metrics with occupation type (n = 305). All retired participants were excluded in this analysis. The ‘no work-related PA’ was excluded since only two participants remained. Figure shows marginal means and standard errors (Z-score). All models were adjusted for sex, occupation type, body fat percentage, seasonality, and level of employment. Age and body fat percentage were modelled using third-degree polynomials. Abbreviations: PA, physical activity.

*Graphical comparison between absolute and relative metrics*

Supplementary Figures 7-9 show adjusted MX plots on the original scales. Supplementary Figure 10 shows adjusted, Z-transformed MX plots without retired participants.

*
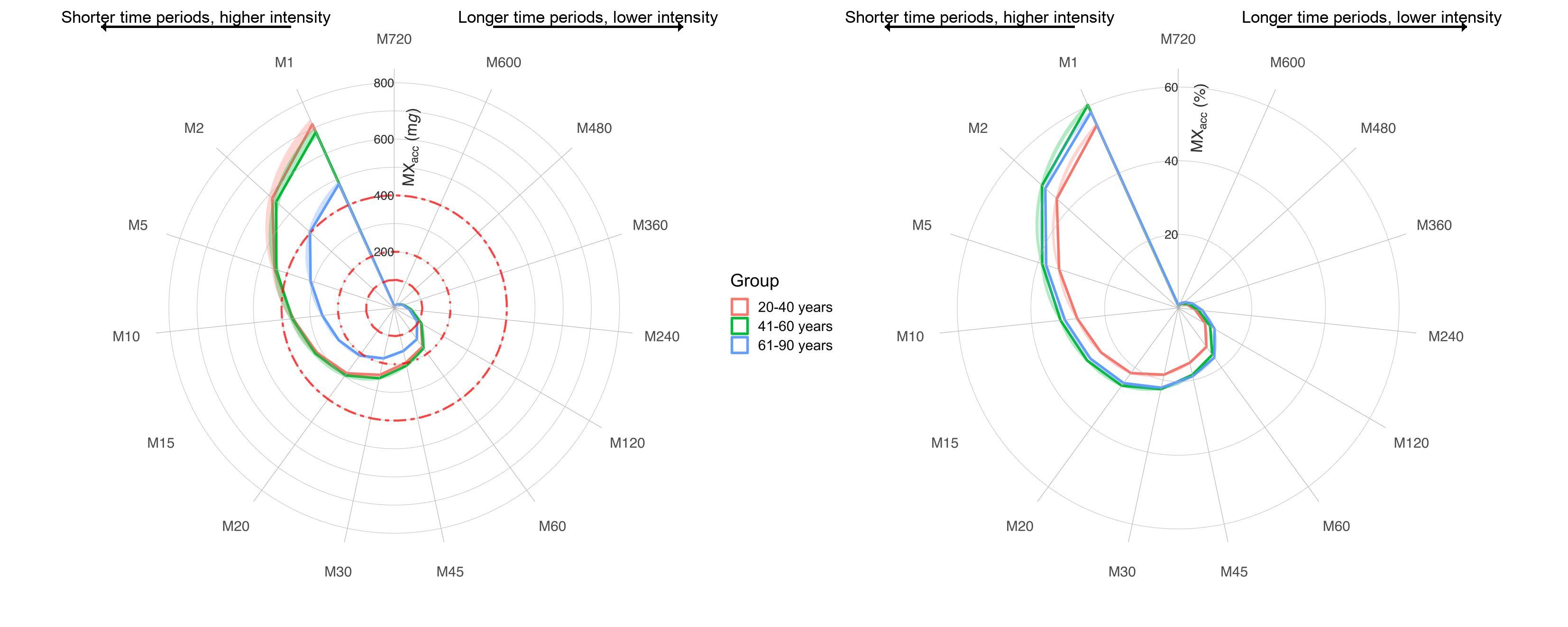
*

**Supplementary Figure 7.** MX plots on the original scale stratified by age group. All metrics were adjusted for age (continuous), sex (dichotomous), body fat percentage (continuous), seasonality (categorical), and degree of employment (categorical). Continuous independent variables were modelled using B-splines with three degrees of freedom. Shading reflects 95% confidence interval of the data. The plot describes the minimum absolute and relative intensities that were accumulated in the most active periods in a 24-h day. Periods range (clockwise) from the most active 720 min (M720; 12 h) to the most active minute (M1). More extended shapes in certain directions indicate more intense physical activity during these periods. The dashed red circles in the left plot reflect slow walking (100 mg), brisk walking (200 mg), and fast walking (400 mg).

*
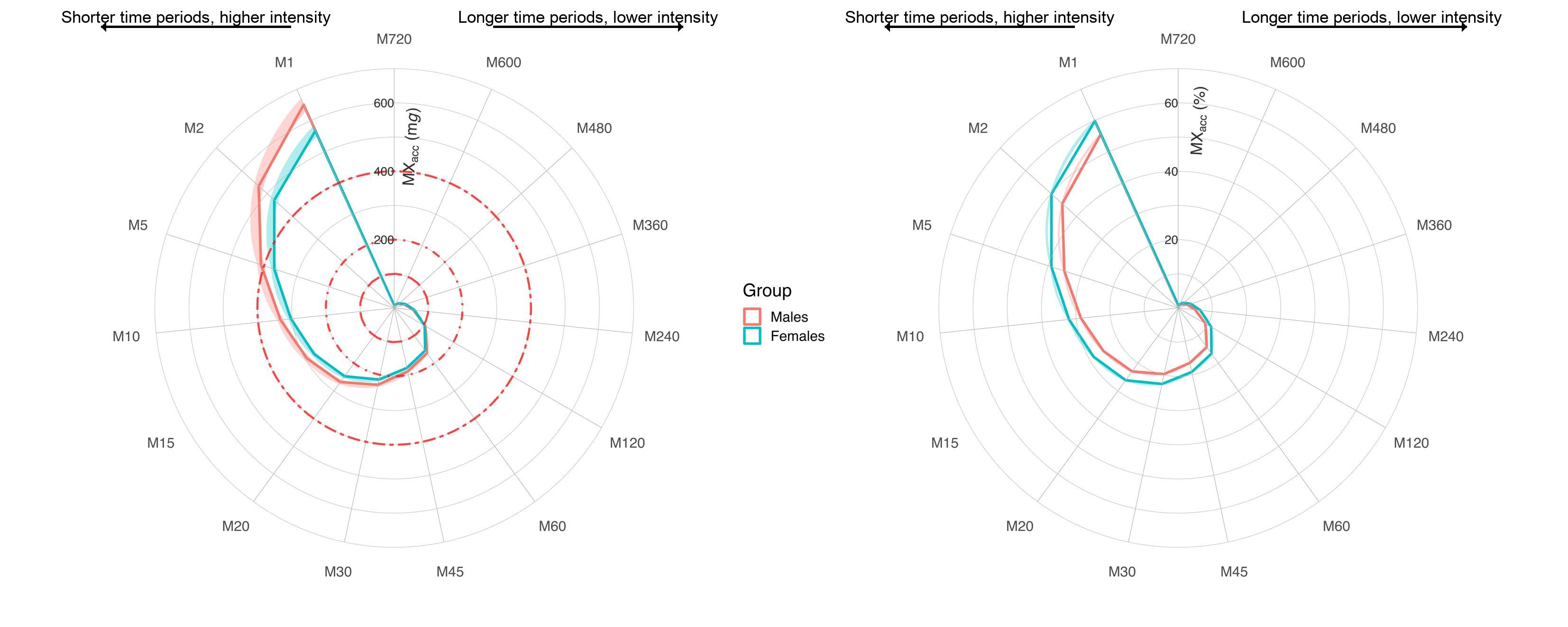
*

**Supplementary Figure 8.** MX plots on the original scale stratified by sex. All metrics were adjusted for age (continuous), sex (dichotomous), body fat percentage (continuous), seasonality (categorical), and degree of employment (categorical). Continuous independent variables were modelled using B-splines with three degrees of freedom. Shading reflects 95% confidence interval of the data. The plot describes the minimum absolute and relative intensities that were accumulated in the most active periods in a 24-h day. Periods range (clockwise) from the most active 720 min (M720; 12 h) to the most active minute (M1). More extended shapes in certain directions indicate more intense physical activity during these periods. The dashed red circles in the left plot reflect slow walking (100 mg), brisk walking (200 mg), and fast walking (400 mg).

*
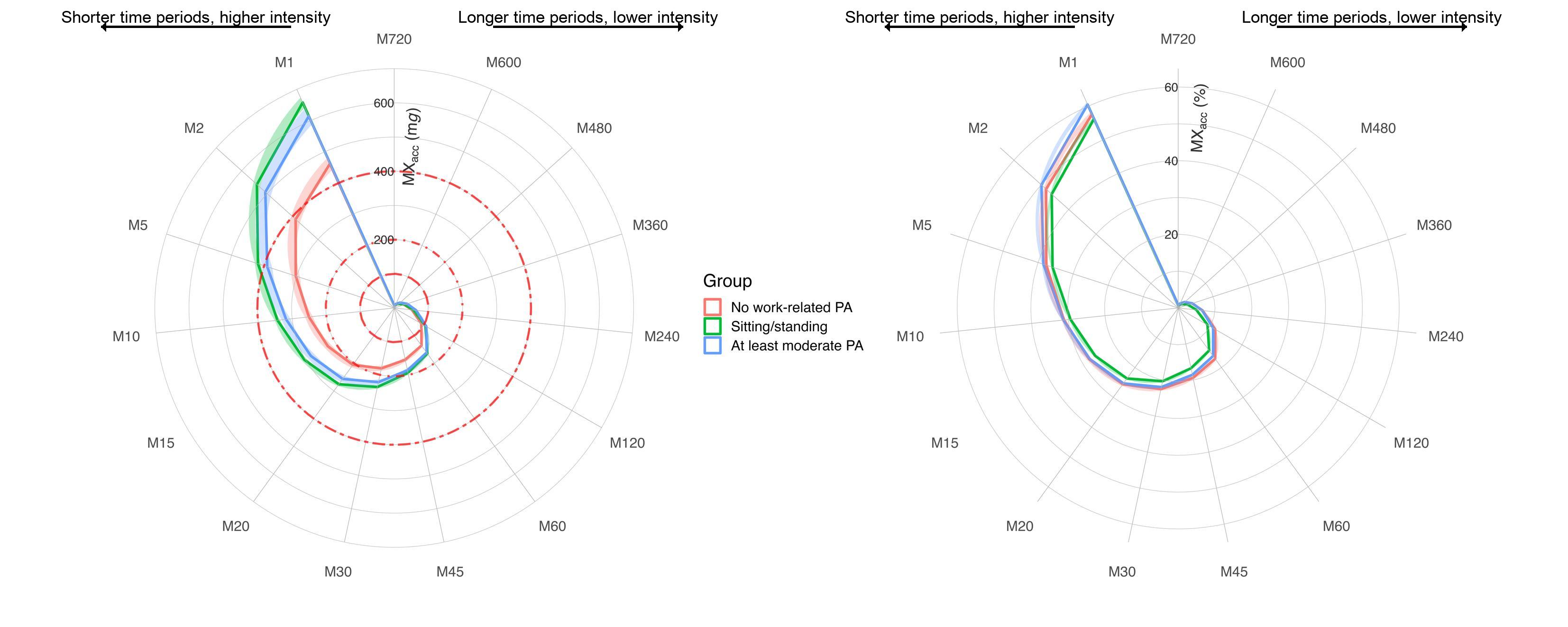
*

**Supplementary Figure 9.** MX plots on the original scale stratified by occupation type. All metrics were adjusted for age (continuous), sex (dichotomous), body fat percentage (continuous), seasonality (categorical), and degree of employment (categorical). Continuous independent variables were modelled using B-splines with three degrees of freedom. Shading reflects 95% confidence interval of the data. The plot describes the minimum absolute and relative intensities that were accumulated in the most active periods in a 24-h day. Periods range (clockwise) from the most active 720 min (M720; 12 h) to the most active minute (M1). More extended shapes in certain directions indicate more intense physical activity during these periods. The dashed red circles in the left plot reflect slow walking (100 mg), brisk walking (200 mg), and fast walking (400 mg).


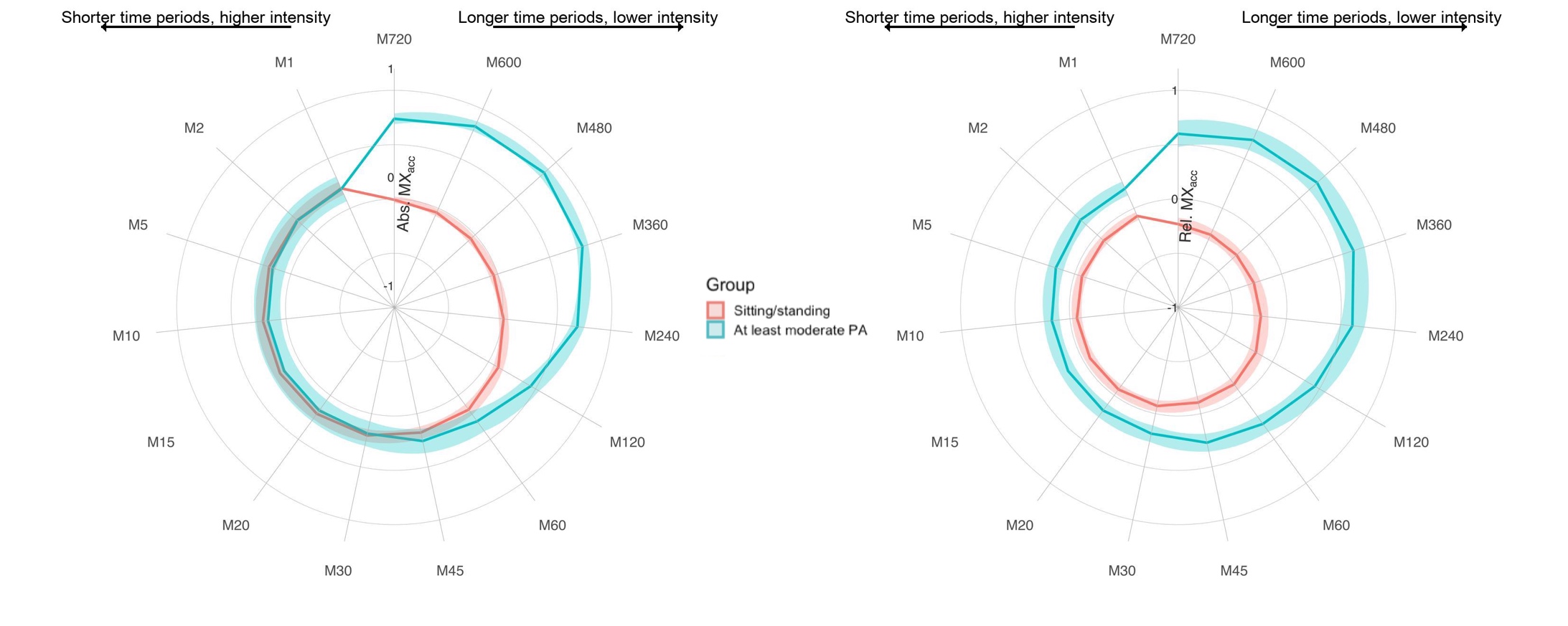


**Supplementary Figure 10.** Adjusted Z-transformed MX plots for absolute intensity and relative intensity, stratified by occupation type (n = 305). All retired participants were excluded in this analysis. The ‘no work-related PA’ was excluded since only two participants remained. All metrics were adjusted for age (continuous), sex (dichotomous), body fat percentage (continuous), seasonality (categorical), and level of employment (categorical). Continuous independent variables were modelled using third-degree polynomials. Shading reflects 95% confidence interval of the data. The plot describes the minimum absolute and relative intensities that were accumulated in the most active periods in a 24-h day. Periods range (clockwise) from the most active 720 min (M720; 12 h) to the most active minute (M1). More extended shapes in certain directions indicate more intense physical activity during these periods.

**REFERENCES**

1. Hildebrand M, van Hees VT, Hansen BH, Ekelund U. Age Group Comparability of Raw Accelerometer Output from Wrist- and Hip-Worn Monitors. Med Sci Sports Exerc. 2014;46(9):1816-24.

2. Jairo HM, Alex VR, Florian H, Séverine S, Vincent TvH. GGIR: A Research Community–Driven Open Source R Package for Generating Physical Activity and Sleep Outcomes From Multi-Day Raw Accelerometer Data. J Meas Phys Behav. 2019;2(3):188-96.

3. Rowlands AV, Edwardson CL, Davies MJ, Khunti K, Harrington DM, Yates T. Beyond Cut Points: Accelerometer Metrics that Capture the Physical Activity Profile. Med Sci Sports Exerc. 2018;50(6):1323-32.

4. Rowlands AV, Fairclough SJ, Yates TOM et al. Activity Intensity, Volume, and Norms: Utility and Interpretation of Accelerometer Metrics. Med Sci Sports Exerc. 2019;51(11):2410-22.

5. Shrier I, Platt RW. Reducing bias through directed acyclic graphs. BMC Med Res Methodol. 2008;8(1):70. doi: 10.1186/1471-2288-8-70.
